# Rare Case of primary Amenorrhea Secondary to Childhood Road Traffic Accident: A Case Report

**DOI:** 10.1007/s13224-021-01429-6

**Published:** 2021-02-18

**Authors:** Shruthi Kesireddy, Niraj Yanamandra

**Affiliations:** 1 Obstetrician and Gynaecologist and Laparoscopic Surgeon, BirthRight By Rainbow Hospitals, Banjara Hills, B-3, Gamut Di Lusso Apartment, Opp To Traffic P.S, Road No 12, Hyderabad, India; 2 Obstetrician and Gynaecologist, Laparoscopic and Hysteroscopic Surgeon, BirthRight By Rainbow Hospitals, Hyderabad, India

**Keywords:** Primary amenorrhea, Pelvic injury, Blunt trauma, Uterine injury

## Introduction

Primary amenorrhea is generally because of congenital defects in the uterine anatomy or because of gonadal dysgenesis. Here we are presenting a rare case of primary amenorrhea due to the uterine transection caused by a blunt trauma secondary to a road traffic accident at the age of 2 years. To the best of our knowledge, this is the only case reported.

## History and Presentation

This patient presented to us at 17 years of age with the complaints of cyclical pain abdomen every month since 2 years, and it was increasing in intensity. Thorough history revealed that she had a history of blunt trauma at 2 years of age due to a road traffic accident leading to a pelvic fracture and bladder injury. She had undergone bladder repair through an emergency laparotomy and she was given conservative management for pelvis fracture. Details of the surgical procedure were not available with them. She developed secondary sexual characteristics at the age of 12 years and at the age of 15 years she consulted a doctor outside for primary amenorrhea.The doctor released labial adhesions and did hymenoplasty, after which she had spotting for 2 days. Surgical notes of that procedure were not available. At 16 years of age, she presented to us with primary amenorrhea and cyclical pain in abdomen. On clinical examination, she had tanners stage 2 thelarche with good axillary and pubic hair development. Per abdomen was soft with a midline scar. Local pelvic examination revealed normal labia and introitus.

USG and MRI revealed normal retroverted uterus with normal cervix and ovaries with a small 1.5 × 1.6 cm hemorrhagic cyst in left ovary. Serum FSH was 14.5 micro-IU/ml with normal female karyotype. Diagnostic laparoscopy and hysteroscopy was suggested to the patient but they got it done outside which revealed old blood in the pelvis, bulky left ovary with a cyst and a normal right ovary with bilateral normal tubes. Uterus was seen completely transected at the lower uterine segment and seen separately from the cervix. Uterus was retroverted with posterior uterine wall adherent to the rectum with flimsy adhesions with normally placed tubes and round ligaments. Hysteroscopy revealed normal vagina and ecto cervix. Endocervical canal was normal with a small endometriotic tissue seen at the upper blind end. 

**Fig. 1:**
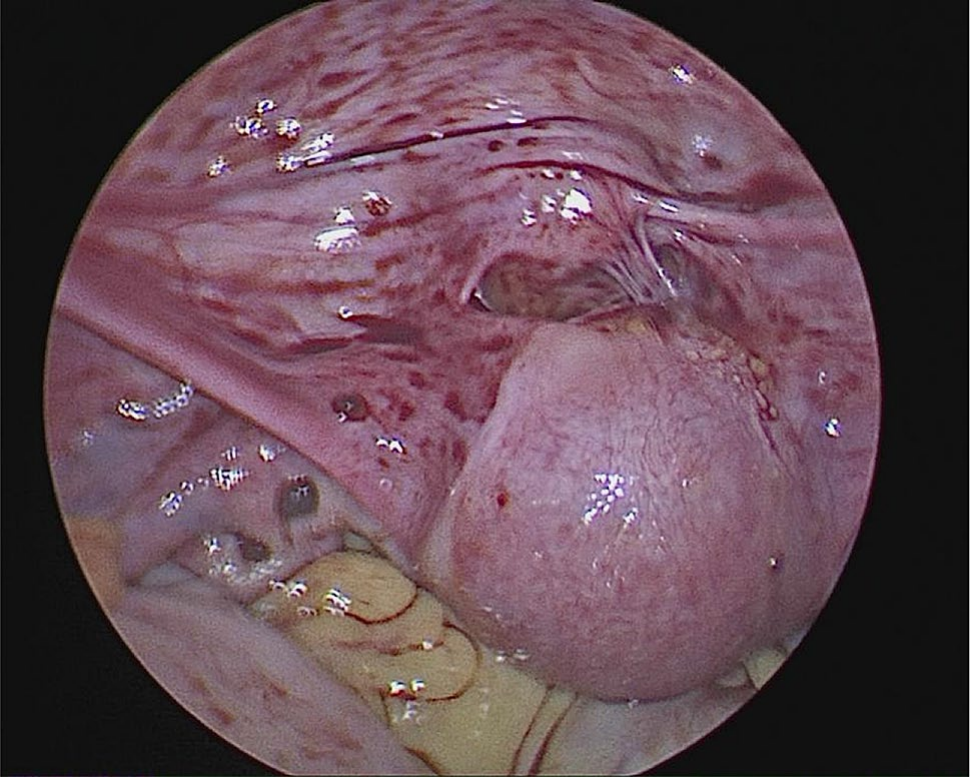
Uterus seen seperated from the cervix

**Fig. 2:**
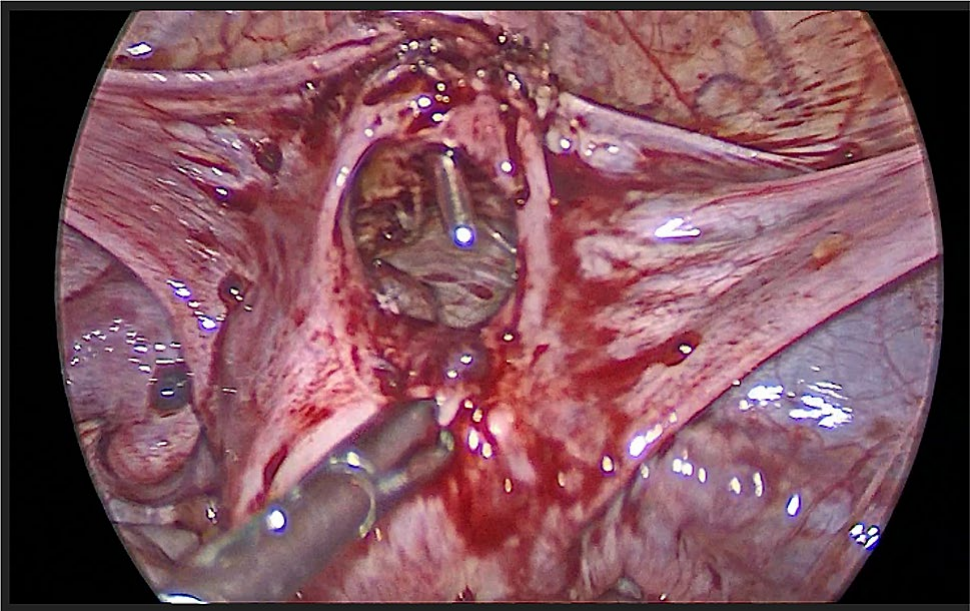
Uterine dilator is guided through the cervix into the uterine cavity for anastomosis

She came back to us for reconstructive uterine surgery. After proper counselling and pre operative evaluation she was posted for laparoscopic reconstructive surgery. Posterior wall of uterine adhesions to rectum were released and the edges of the lower uterine segment and cervix were freshened after the bladder was pushed down slightly (Fig. 1). Through the vagina a uterine sound was passed through the cervix and under vision the upper blind end of the cervix was pierced and then the uterine sound was guided through the uterine cavity( Fig. 2). Four anastomotic sutures were taken separately with the 1–0 vicryl on four sides uniting the uterine lower segment and the upper part of cervix. Haemostasis was secured. 12 F foleys catheter was placed inside the uterine cavity and inflated with 4 cc distilled water to maintain the patency of the anastomotic area. It was left for 3 weeks and then removed. She was discharged after 1 day. Oral contraceptive pills were given continuously for 63 days to avoid bleeding during the healing process. She had her normal period after 70 days of the procedure. Since then (i.e., 1 year), she is regularly getting periods without any cyclical pain.

## Discussion

Bladder in small children is mostly intraabdominal and is less protected by pubic symphysis. If any blunt injury occurs to the abdomen with full bladder, the bladder is most likely to rupture intraperitoneally [1]. Blunt abdominal injury in a female child causing pelvic fracture, is generally protective to urethra and uterus due to the structural anatomy and underdeveloped uterus [2]. The most plausible explanation for uterine injury in this case could be either due to the direct injury leading to complete transection at the lower uterine segment which was missed at the time of bladder repair as it was very small uterus at that time or it could be due to the iatrogenic cause while the bladder was repaired.

Evaluation of primary amenorrhea should always start with secondary sexual characteristics development which indicates that her ovaries are normal. In this case, ovaries are well developed along with uterus and cervix as visualised on the scan. As her Ser FSH was 14.5, karyotype was also done which showed normal female karyotype. Generally we suspect genital tuberculosis [3] in cases of primary amenorrhea with normal anatomy and normal karyotype. In this case, cyclical pain every month with normal endometrial echo,indicated that her endometrial activity is good which generally rules out tuberculous endometritis. In view of history of bladder repair in the childhood, we had a high suspicion of local pelvic pathology which can be the cause of primary amenorrhea.Probably there was perineal laceration at the time of road traffic accident which required labial adhesiolysis and hymenoplasty.As there was minimal endometriotic tissue seen in the blind upper end of the cervix, she had spotting per vaginum for 2 days after the labial adhesiolysis. As the endometrial cavity was normal, she was having retrograde menstruation leading to endometriosis. Systematic evaluation of primary amenorrhea revealed normal uterus and ovaries with good sexual development with normal female karyotype. This lead to the decision of doing a diagnostic laparoscopy which revealed this rare uterine injury. 

Following basic surgical principles with good anastomoses of uterus to the cervix helped the patient to have a good approximation leading to regular periods without cyclical pain and preventing endometriosis. She was advised to go for an elective cesarean section in the future.

## Conclusion

Uterine injury as a cause of primary amenorrhea must be suspected when the girl presents with primary amenorrhea with a background of road traffic accident leading to bladder repair in childhood specially when the secondary sexual characters and uterus are normal. It is important to look at the genital structures specially uterus in a female child, while repairing the bladder injury caused due to blunt trauma.Systematic evaluation and good surgical principles are needed to treat the patient at the right time and retain her future fertility.
